# Evaluation of Prognostic Factors for Palatal Fistulae after Cleft Lip and Palate Surgery in a North-Western Romanian Population over a 10-Year Period

**DOI:** 10.3390/ijerph18147305

**Published:** 2021-07-08

**Authors:** Daiana Antoaneta Opris, Horia Opris, Cristian Dinu, Simion Bran, Grigore Baciut, Gabriel Armencea, Ileana Mitre, Horatiu Alexandru Colosi, Mihaela Baciut

**Affiliations:** 1 Department of Maxillofacial Surgery and Implantology, Iuliu Hatieganu University of Medicine and Pharmacy, 400012 Cluj-Napoca, Romania; daiana.a.opris@gmail.com (D.A.O.); horia.opris@gmail.com (H.O.); dr_brans@yahoo.com (S.B.); gbaciut@umfcluj.ro (G.B.); garmencea@gmail.com (G.A.); ilmitre@yahoo.com (I.M.); mbaciut@umfcluj.ro (M.B.); 2 Department of Medical Education, Division of Medical Informatics and Biostatistics, Iuliu Hatieganu University of Medicine and Pharmacy, 400012 Cluj-Napoca, Romania

**Keywords:** cleft palate, fistula, epidemiology, oro-nasal communication

## Abstract

Cleft lip and palate is the most frequent birth anomaly, with increasing reported rates of complications, such as palate fistulae. Current studies concerning the occurrence rate of cleft lip and palate (CLP) report 2 to 10 cases in 10,000 births. The purpose of this study was to investigate the existence of factors that could predict the occurrence of fistulae after cleft lip and palate surgery. A retrospective study was performed by collecting and analyzing data from all patients who were operated for cleft lip and/or palate in the Maxillo-Facial Department of the Emergency Clinical County Hospital of Cluj-Napoca, Romania, between 2010 and 2020. We investigated the existing evidence for possible links between the number of fistulae observed after the primary palatoplasty and the age at which the primary palatoplasty was performed, the sex of the patient, the type of cleft, the timing of the surgical corrections, and the presence of comorbidities. A total of 137 cases were included for analysis. A significant link between the number of fistulae and the type of cleft was found (with fistulae occurring more frequently after the surgical correction of CLP—*p* < 0.001). No evidence was found for the existence of significant links between the number of fistulae and the patient’s sex, the timing of surgery, or the presence of comorbidities. This study concluded that the incidence of palatal fistulae appears to be influenced by the type of cleft (CLP), but not by the sex of the patient, the timing of surgery, or the presence of comorbidities.

## 1. Introduction

Orofacial clefts (OFCs) are the most common congenital malformations of the craniofacial region and can occur solely or can be associated with additional abnormalities or genetic syndromes [1,2].

The prevalence in Europe, based on the International Perinatal Database of Typical Oral Clefts (IPDTOC), is 9.92 in 10,000 births for OFCs, with cleft lip seen in 3.28 per 10,000 births and cleft lip and palate (CLP) in 6.64 per 10,000 births [1].

When referring to clefts involving the palate, such as cleft lip and/or palate (CL/P) or cleft palate only (CP), the success of the treatment is quantified considering the postoperative development of palatal fistulae, velopharyngeal insufficiency, phonation disorders, and alterations in the mid-face growth pattern. The main objective of palatoplasty is creating the optimal anatomical conditions for normal speech development, assisted by logopedics, and for the appropriate growth of the mid-face [1].

The patency between the oral and nasal cavities, known as palatal fistulae, is a common complication after cleft palate surgery, with an incidence of 4–45% and high recurrence rate, ranging from 37–50%, even in the best hands of experienced surgeons [3,4,5].

The prognostic factors of palatal fistulae, severity of the cleft, cleft width, and type of cleft have been taken into consideration in recent studies [6]. Surgical techniques, such as single- or two-stage palate repair, and surgeon-specific factors were also considered to influence the rate of fistula occurrence. Other factors, such as flap design, tension-free closure, hematoma formation, and local septic conditions, may also lead to this complication [4].

The most common complications and inconveniences of palatal fistulae are hypernasality or rhinolalia aperta, backflow of fluids through the nose, as well as the impossibility of bone grafting while oro-nasal fistulae are still present [7]. The timing of the repair is dependent on the degree of disturbance, especially in speech, and should be corrected as soon as possible. A communication defect that leads to significant regurgitation into the nose also needs to be quickly addressed by a secondary palatal surgical intervention. Speech evaluation is essential and may be an indication regarding early repair of fistulae. Planning in these secondary interventions, along with other surgical repairs, such as those targeting velopharyngeal insufficiency, alveolar bone graft, or lip revision, is a factor to be taken into consideration [8].

The Pittsburgh Fistula Classification system has been developed as a standardized classification for palate fistulae [3], and surgical protocols have been established using this assessment [9].

Even though palatoplasty is a one-time intervention, the auxiliary treatments, including speech therapy, orthodontics, and management of possible complications, are a long-term treatment that is often incomplete, even in young adults [7]. The child born with a cleft lip and/or palate also requires a multidisciplinary approach of different specialties in order to improve the overall results of the treatment [10].

This long course of treatment, involving a multidisciplinary team, as well as the increased number of interventions, such as subsequent lip corrections or secondary plastic interventions for closing the palatal fistulae, often represent a psycho-social and financial burden for both the families and the healthcare system.

In order to evaluate which factors, such as age at the time of the intervention, sex, type of cleft, or presence of comorbidities, may influence the number of secondary surgical interventions after the primary palatoplasty, we designed and performed a 10-year, retrospective study in a large maxillofacial surgery center from north-western Romania.

## 2. Materials and Methods

The study protocol was evaluated and approved by the Research Ethics Committee of the Iuliu Hațieganu University of Medicine and Pharmacy from Cluj-Napoca, Romania (approval number 277 from 11 August 2020), allowing the collection of data from the Maxillofacial Department of the Emergency Clinical County Hospital of Cluj-Napoca. All personal information was anonymized, and the process was compliant with the current General Data Protection Regulation GDPR 2016/679. The electronic database of the hospital was accessed to collect the necessary data.

### 2.1. Inclusion Criteria

In this study, all the patients from the Maxillofacial Department of the Emergency County Hospital of Cluj-Napoca, Romania, treated for CLP or CP between January 2012 and December 2020, of all ages and both genders, were included.

The International Classification of Diseases (ICD-10) diagnosis codes used in the study were Q35 (cleft palate) and Q37 (cleft palate and cleft lip).

All the studied patients were operated on by two consultants, using von Langenbeck, Veau–Wardill–Kilner, and Bardach palatoplasty.

### 2.2. Exclusion Criteria

All patients who did not undergo all the surgical steps in our clinic, and patients with facial clefts but without palatal clefts, as well as any oro-nasal communications that were not cleft related, were excluded. Patients with intentionally unrepaired fistulae of the primary and secondary palate were also excluded. All cases with incomplete data were also excluded.

### 2.3. Data Analysis

A unique identifier was attributed to each patient. From the clinical record of each patient, the following pieces of information were extracted and coded as study data: diagnosis, sex, type of cleft, number of admissions, date of admission, type of surgical intervention, presence of comorbidities, and age at primary and secondary palatal surgery.

Data were collected using Microsoft Excel (Microsoft Corp., Redmond, WA, USA) and analyzed using IBM SPSS Statistics for Windows, Version 25.0 (IBM Corp., Armonk, NY, USA).

The normality of the collected data was investigated using Q-Q plots and Shapiro–Wilk normality tests. Due to the asymmetrical distribution of the number of secondary palatal surgical interventions, hypotheses were tested using Mann–Whitney tests for independent samples and confirmed using Fisher’s Exact tests in the qualitative approach of the same hypotheses, based on 2 × 4 contingency tables.

Two-tailed significance *p*-values were computed and interpreted, with the level of statistical significance chosen as α = 0.05.

Descriptive statistics were calculated and reported as medians and interquartile ranges (IQR). Box and whiskers charts, as well as column charts, were plotted to illustrate these descriptors and to illustrate the tested hypotheses.

Four main hypotheses were tested, concerning the existence of a link between the number of secondary palatal surgical interventions and the age at primary palatoplasty, comorbidities, sex, and type of cleft.

## 3. Results

**Figure 1:**
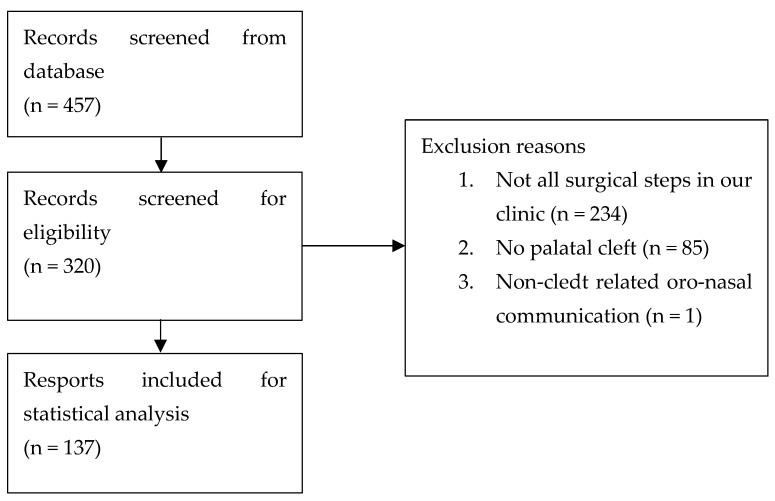
Summary of the patient records included in the study.

In total, 457 patients were included in the preliminary database search. After screening the results, only 137 patients were included in the statistical analysis of this study, as illustrated in Figure 1.

**Figure 2:**
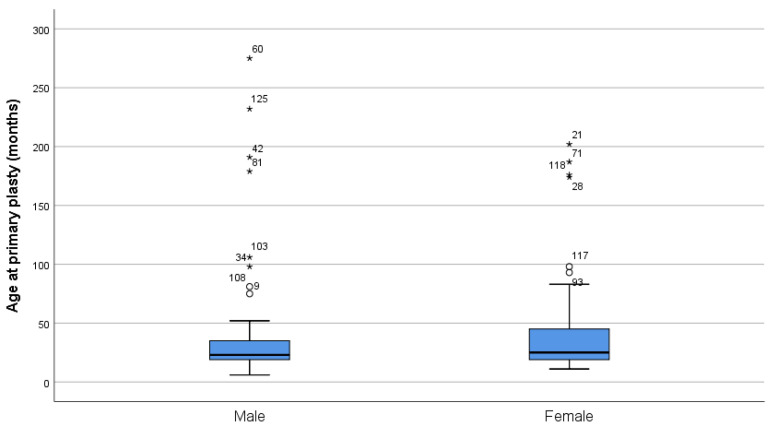
Ages of the male and female patients at their primary surgical intervention. “*”—outliers.

Male and female patients in the studied sample underwent their primary surgical palatal intervention at comparable ages (*p* = 0.938—Mann–Whitney test), as presented in Table 1 and illustrated in Figure 2.

The prevalence of fistulae found in the studied sample was 29 out of 137 cases, corresponding to 21.17% (95% CI 15.16–28.75%).

The results obtained after testing the investigated hypotheses, regarding the prognostic factors of the recurrent fistulae and subsequent secondary palatal surgical interventions, are summarized in Table 2.

After testing the above null hypotheses, only the last one, concerning a potential link between the type of cleft and the number of secondary palatal surgical interventions, could be rejected, using both the quantitative (Mann–Whitney) and categorical (Fisher’s Exact) approach (*p* < 0.001).

The detailed hypotheses and their corresponding descriptive elements are presented below.

### 3.1. Age

The null hypothesis that the number of palatal fistulae repairs did not differ based on the age of the patient being <4 years (N = 109) vs. >4 years (N = 28) at the moment of the primary surgical palatal intervention could not be rejected (*p* = 0.982—Mann–Whitney test; *p* = 1.000—Fisher’s Exact test).

**Figure 3:**
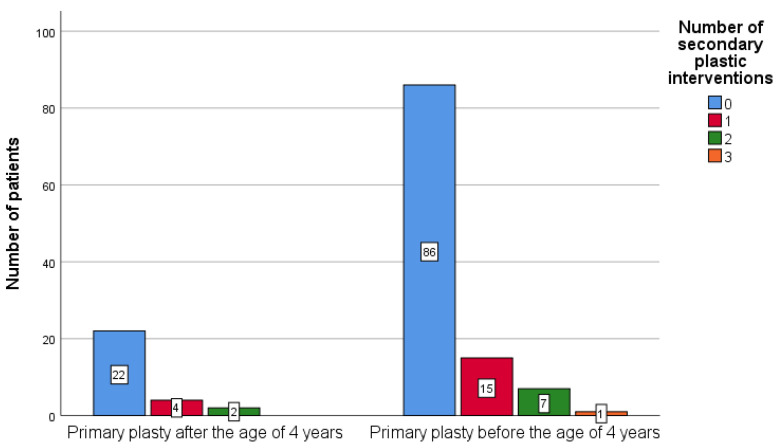
Frequency of secondary palatal surgical interventions in patients younger and older than 4 years.

The median for both groups was 0, since most of the patients, in both groups, underwent no secondary plastic interventions, as can be observed in Figure 3. Only one patient, who had the primary palatal surgical intervention before 4 years of age, had three secondary surgical palatal corrections.

### 3.2. Comorbidities

The null hypothesis that the number of secondary palatal repair surgical interventions did not differ significantly between patients with (N = 80) and without comorbidities (N = 57) could not be rejected (*p* = 0.316—Mann–Whitney test; *p* = 0.585—Fisher’s Exact test). This study did not find evidence of a significant link between the presence of comorbidities and the number of palatal surgical interventions.

**Figure 4:**
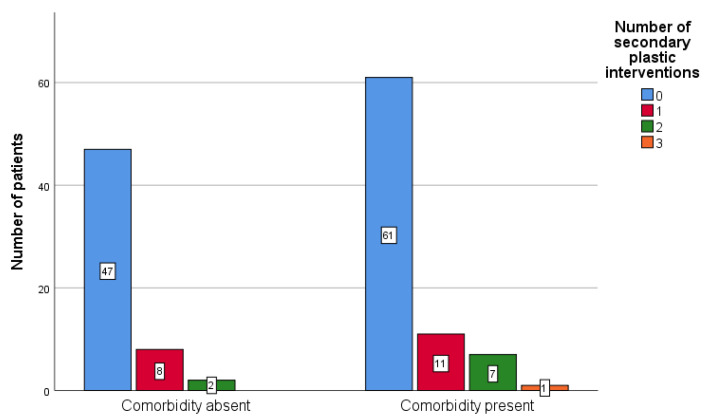
Frequency of secondary palatal surgical interventions based on the presence of comorbidities.

Figure 4 illustrate that the median of both groups was 0, meaning that more than half of the patients in each group had no plastic surgical correction. Only one patient had three surgical palatal corrections and can be seen in the group with existing comorbidities.

### 3.3. Sex

The null hypothesis that the number of secondary palatal repair surgeries was not influenced by the sex of the patient (N = 65 male vs. N = 72 female patients) could not be rejected (*p* = 0.356—Mann–Whitney test; *p* = 0.286—Fisher’s Exact test).

**Figure 5:**
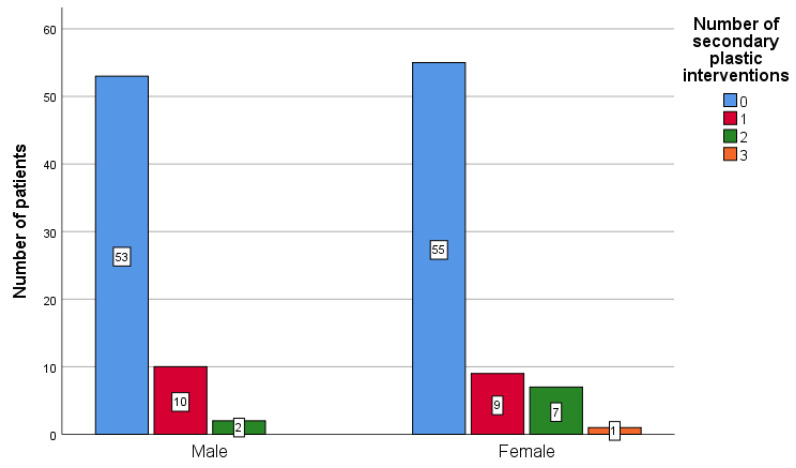
Frequency of secondary palatal surgical interventions based on the sex of the operated patients.

The median number of secondary interventions was 0 in both male and female patients (Figure 5), since most patients had no secondary palatal plastic correction. Patients of both genders who did have secondary plasty can also be observed, with one female patient having three surgical site corrections.

### 3.4. Type of Cleft

The null hypothesis that the number of secondary surgical palatal repairs did not differ based on the type of clefts, CP only (N = 63) vs. CLP (N = 74), was rejected, based on the calculated two-tailed *p* < 0.0001, in both the Mann–Whitney and Fisher’s Exact tests. Therefore, the number of secondary palatal surgical repairs differed significantly between patients suffering from CLP, compared to patients with CP only.

**Figure 6:**
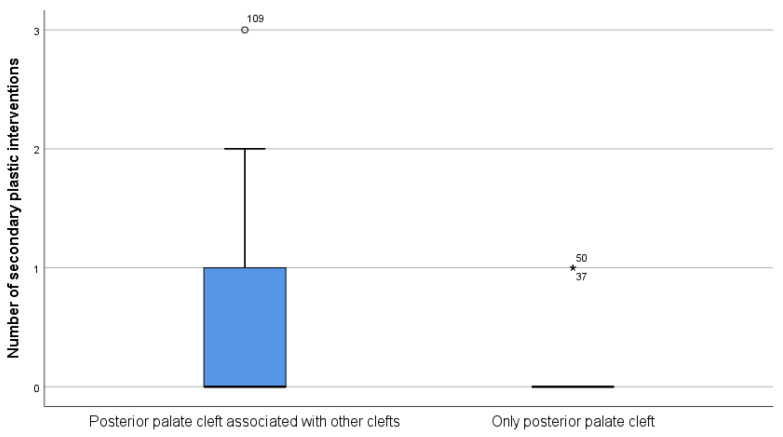
Box and whisker plots comparing the number of secondary palatal surgical interventions based on the type of cleft. “*”—outliers.

**Figure 7:**
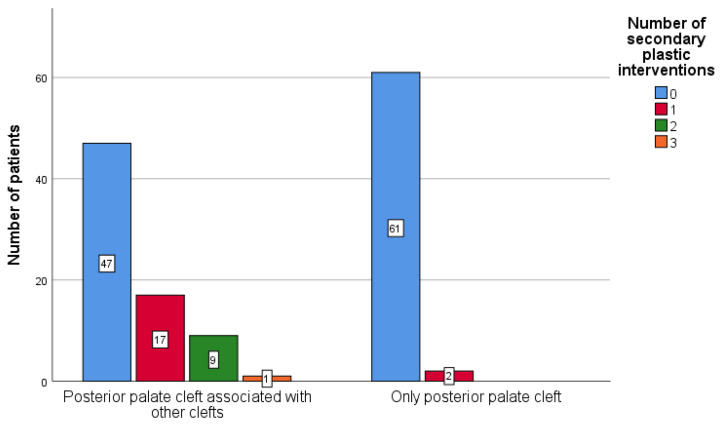
Frequency of secondary palatal surgical interventions based on the type of cleft.

Figure 6 and Figure 7 present the distribution of the number of secondary palatal surgical interventions depending on the type of cleft. Although the median value for both groups was zero, and thus more than half of the patients in both groups had no secondary palatal repairs, 36.5% (27 patients) of the 74 patients suffering from CLP had one or several secondary palatal surgical interventions, compared to 3.2% (2 patients) of the 63 patients suffering from CP-only, who also needed a secondary palatal surgical intervention for the repair of a palatal fistula.

## 4. Discussion

The current study has reached its aim, by investigating the possible prognostic factors for the occurrence of palatal fistulae following the primary palatal plasty in patients with cleft palate (CP). Among the investigated possible prognostic factors (age, sex, comorbidities, and type of cleft), the coexistence of other clefts along with a CP was the only factor found to be prognostically linked to the occurrence of palatal fistulae and to the need to perform one or several secondary surgical fistula repairs.

### 4.1. Strengths and Limitations of the Study

To the best of our knowledge, this is the first study, performed in Central and Eastern Europe, to investigate the prevalence and prognostic factors of fistulae occurring after the primary closure of the CP and CLP. We investigated 457 patients treated for this pathology in a single center, over a span of 10 years. Owing to the high levels of experience and fairly similar surgical skills of two consultant surgeons, we included both of their patients in this study, thereby being able to investigate a fairly large database, capable of yielding good precision to the results of our investigation. This study is one of the few studies published worldwide that tried to investigate the possible predictors of fistula occurrence after cleft palate surgery.

Nevertheless, the design of this study as a retrospective single-center evaluation left it subject to many unavoidable limitations of retrospective appraisal: the available data were limited by the type of information collected for clinical use. The included patients were treated in a single surgical center, the collected data included only surgical aspects of the interventions, and no cast models and no dimensions of the cleft in relation to the size of the maxilla were recorded in a consistent way that could have been used for research purposes. Furthermore, data regarding the coexisting orthodontic treatments could not be assessed as part of this study. No uniform grading and scaling of the severity of the cleft was used. The surgical technique was not noted specifically for each patient although our center has a clear protocol for each step of the management of the cleft patient, and no surgical modifications or difficulties were noted in the medical records of all patients. The size of the oro-nasal communication defect was not recorded, and a number of codification errors were also found and corrected throughout the available data. The study had no available data concerning the speech and phonation difficulties of the patients and the degree of impairment due to the fistula. The study could also not include prenatal data regarding the conditions of birth and pregnancy specific for each cleft patient. Some patients may have developed fistula but were lost from clinical follow-up. Finally, other patients may have moved to other surgical centers and were also not included in this study.

### 4.2. Comparison with Other Studies

Regarding the timing of the primary palatoplasty, there has been a long debate about early versus late repair. Early repair (patients younger than 6 months) has been shown to have a higher incidence of fistula formation (*p* = 0.0026) [10]. Velopharyngeal insufficiency (VPI) was not significantly different between early or later repairs. The occurrence of postoperative fistulae has been linked in the published literature to a higher incidence of VPI [11].

A recent study [11] concerning the prevalence and etiology of palatal fistulae reported an incidence of 9.6%, mostly after closure with local and buccal flaps. The rate of fistula recurrence was 18.2%. The same study found that complete clefts that involved both the primary and the secondary palate lead to more oro-nasal communications [12].

Another study [12], performed on 129 consecutive non-syndromic patients, revealed an incidence of fistulae after palate closure of 23%. The type of palate closure influenced the frequency of fistulae: 10% (Furlow), 22% (von Langenbeck), and 0% (Dorrance). Age did not significantly influence the occurrence rate of fistulae found in that study. The experience of the surgeon performing the initial closure had a significant effect. Of all patients included in that study, 37% developed recurrent cleft fistulae, but this was not influenced by the severity of the cleft or by the type of primary repair [13].

A study [13] performed on 103 consecutive non-syndromic cleft patients found a 33% rate of fistulae recurrence. The incidence was significantly higher for Veau type III and IV clefts compared to Veau I and II clefts (*p* = 0.0441). The study revealed no link between the fistulization rate and the operating surgeon, the sex of the patients, their age at primary palatoplasty, the type of palatoplasty, and the use of pre-surgical orthopedics or palatal expansion [14].

Reviewing Furlow’s palatoplasty by a single center revealed a 9.7% incidence of fistulae at 3 months postoperative, in a 62-patient retrospective analysis. The width of the cleft was linked to the incidence of postoperative relapse (*p* = 0.001) and oro-nasal fistulae (*p* = 0.011). The incidence rates were positively correlated with the width of the cleft when it exceeded 6.8 mm and 7.5 mm. Thus, Furlow’s repair appeared not to be recommended for patients with wide clefts [15].

The width of the cleft is often times cited by authors as an indicator of cleft severity, but until now there is no consensus on this matter regarding the anatomical landmarks for measurement. It may be considered the distance between the two palatal shelfs in the intertuberosity region [16], or the distance between the curved vomer and the contralateral palate shelf, referred to in the literature as the “true cleft” [17,18]. In our center, we use a tension-free suture type of palatal primary cleft repair.

In the literature, specific microbiological factors have been frequently cited as a factor of complications in cleft lip and palate [19,20] and in our center we screen every single patient before admission and surgical treatment.

Another recent study has tried using acellular dermal matrix to help repair palatal fistulae in 20 consecutive patients, with a success rate of 85% [21].

The results of our study concur with the evidence found by a systematic review of the scientific literature [16], suggesting a statistically significant (*p* = 0.03) higher incidence of fistulae for CLP (17.9%) compared to CP (5.4%). That review found no difference in the incidence of fistulae depending on the continent and technique [22]. Another meta-analysis reported an incidence of 4.9% of palatal fistulae, with Veau type IV being directly linked to the occurrence of palatal fistulae (*p* < 0.001) [23].

A further study [18] reported an incidence of palatal fistulae as low as 2.4% after primary repair in Veau type III and IV clefts. In that study, the cleft width/cleft-to-total-width of the palate ratio was associated with the occurrence of fistulae. Other investigated factors, such as syndromes, age, and adoption status, were not. Most complications were attributed to surgical decision and technical difficulties [24].

Other researchers [19], trying to find prognostic factors of palatal fistulae in cleft lip/palate cases, found such combined clefts to be associated with a wider mean cleft and a higher incidence of shorter palate than in cases of cleft palate only. Velopharyngeal insufficiency was more frequent in CLP, male patients, greater cleft widths, and shorter palates [25]. Other studies reported a fistulization rate of 5.5% and no identifiable association with the type of cleft or the use of an acellular dermal matrix [26].

An even more recent systematic review and metanalysis revealed that Furlow’s technique was less prone to postoperative fistula occurrence. One-stage repairs were also linked to fewer fistulae and fewer velopharyngeal insufficiency occurring, compared to two-staged repairs [27].

The recommended closure of the clefted palate is at the age of around 10–14 months [28,29]. The results of this study reveal the median value for the primary palate surgical intervention to be 41.22 months for male patients and 40.75 months for female patients. Due to socio-economic factors, often, we cannot schedule the surgical interventions in the due time. Frequently, these patients have a late diagnosis. For many of them, pharyngeal streptococcus infections or respiratory intercurrences occur, and we are forced to reprogram them. Many of them are institutionalized and it is very hard to synchronize and prepare these patients for surgery.

Considering the heterogeneity of evidence that has resulted from single-center, retrospective studies so far, a justified need exists to develop and implement a collaborative study protocol for a multi-center, prospective investigation of the prognostic factors involved in the occurrence of fistulae and other complications, such as velopharyngeal insufficiency, after the primary closure of palatal clefts.

## 5. Conclusions

Based on the results of this single-center experience over 10 years, the current study suggests that the number of secondary palatal fistulae is linked to the type of cleft, being more frequent in cases of CLP, compared to CP alone. There appears to be no significant influence of age at primary surgery, sex, or presence of comorbidities, on the occurrence of secondary palatal fistulae and the number of secondary palatal repairs.

A prospective, multi-center study should be planned and performed in order to further clarify the role of prognostic factors in the occurrence of palatal fistulae after the primary closure of palatal clefts.

## Figures and Tables

**Table 1:** Ages of the male and female patients at their primary surgical intervention.

	Male		Female		2-Tailed *p*-Value
	Median (IQR)	Mean (SD)	Median (IQR)	Mean (SD)	
Age at primary surgical intervention (months)	23 (18)	41.22 (50.41)	25 (27)	40.75 (40.57)	0.938

**Table 2:** Summary of the tested hypotheses regarding the number of secondary palatal surgical interventions.

Criteria	Evaluation of Criteria	2-Tailed *p*-Value (Fisher’s Exact Test)	2-Tailed *p*-Value (Mann–Whitney Test)
Age	<4 years vs. >4 years	>0.05	>0.05
Comorbidities	Presence/Absence	>0.05	>0.05
Sex	Male/Female	>0.05	>0.05
Type of cleft	CP/CLP	<0.001	<0.001

## Data Availability

Data available on request due to privacy and ethical restrictions.
